# Effects of Oral Levamisole as an Adjuvant to Hepatitis B Vaccine in HIV/ AIDS Patients: A Randomized Controlled Trial

**DOI:** 10.5812/hepatmon.6234

**Published:** 2012-09-30

**Authors:** Babak Sayad, Seyyed Moayed Alavian, Farid Najafi, Bita Soltani, Maria Shirvani, Alireza Janbakhsh, Feyzollah Mansouri, Mandana Afsharian, Siavash Vaziri, Arash Alikhani, Homayoon Bashiri

**Affiliations:** 1 Liver Disease and Hepatitis Research Center, Kermanshah University of Medical Sciences, Kermanshah, IR Iran; 2 Research Center for Gastroenterology and Liver Diseases, Baqiyatallah University of Medical Sciences, Tehran, IR Iran; 3 Health Research Center, Kermanshah University of Medical Sciences, Kermanshah, IR Iran

**Keywords:** HIV, Hepatitis B Virus, Vaccination, Levamisole

## Abstract

**Background:**

Human immunodeficiency virus (HIV) infected patients are also frequently exposed to the hepatitis B virus (HBV), due to the common routes of transmission, therefore, prevention of hepatitis B results in decreased complications of the disease.

**Objectives:**

Since the immune response of HIV patients to hepatitis B vaccination is less robust than that found in healthy individuals, this study aimed to evaluate the effect of a levamisole adjuvant on increasing the immune response.

**Patients and Methods:**

In this study, 89 HIV infected patients, without a history of HBV infection or vaccination, were randomly allocated into experimental (44 patients) and control (45 patients) groups. HBV vaccination was performed using the Hepavax-Gene TF vaccine, 40 μg three times at intervals of; zero, one, and three months. Levamisole 50 mg twice a day or a placebo, was administered to the experimental and control groups, respectively, for a period of six days before to six days after the vaccination. Immune response was evaluated by measuring hepatitis B surface antibodies (HBsAb) concurrently with the second and third vaccine administration, and at one and three months at the conclusion of the vaccination program.

**Results:**

The immune response following the threevaccinations was higher in those who were receiving levamisole compared with the controls (90% vs. 65.38%) (P = 0.05). Furthermore, the immune response and the mean antibody titer following the repeated vaccination in the experimental group showed a higher increase than in the control group. The immune response and the mean titer of antibody were not associated with; age, sex, body mass index, history of smoking and/or intravenous drug use in either of the groups. However, regarding CD4+ cells more than 200 cell/mm3, mean antibody production significantly increased in both groups.

**Conclusions:**

Using levamisole with the hepatitis B vaccination can increase the immune response and antibody titer mean in HIV infected patients. Since these patients have a more complete response with CD4+ cells more than 200 cell/mm3, vaccination and effective adjuvants seem to be most beneficial when CD4+ cells are greater than 200 cell/mm3, in HIV infected patients.

## 1. Background

Human immunodeficiency virus (HIV) infected patients are often vulnerable to developing hepatitis B as well, this is due to their common routes of transmission (1, 2). In Asia, 34% to 98% of HIV infected cases show evidence of a previous hepatitis B infection (3). Moreover, HIV infection has a significant effect on the course of a hepatitis B virus (HBV) infection, causing a three-fold increase in the incidence of permanent antigenemia with hepatitis B surface antigens (HBsAg). Liver-related mortality rates of patients with both HBV and HIV infections show a 10-15 fold increase compared with those with only one of these infections. Also, it has been observed that HIV infected patients who have previously been infected with HBV were more likely to develop chronic disease (4, 5) which can result in; cirrhosis, hepatic failure and development of hepatocellular carcinoma (3, 6). Therefore, prevention of hepatitis B in HIV infected patients plays an important role in improving the quality and quantity of their lives. It should be noted that more than 95% of the general population with a normal immune system will have an appropriate response to the standard vaccine dose (2, 7, 8), however, the immune response in HIV infected patients is reduced, estimated to be about 40% to 60% (2, 9). This results from the development of the infection and the reduced number and function of CD4+ lymphocytes, as well (10). Regarding the aforementioned issues and the importance of effective vaccination in these patients, many studies have been conducted in order to find more effective methods, for example using intradermal injections instead of intramuscular injections and administering these once a week (11), at double doses (12) or higher doses (13, 14) instead of the usual dose and using adjuvants with the vaccination (1, 9). Levamisole is one of the adjuvants which have been used widely in the treatment of worm-borne diseases. Since 1972 when its stimulatory effect on the immune system was discovered, it has been used for many other diseases as well. This drug can improve the immune system by stimulating macrophage and T cell functions (15, 16). Several studies have used this stimulatory effect, in order to increase immunization following vaccination, thus leading to more effective results (16-18).

## 2. Objectives

The present study aimed to evaluate the effect of levamisole, as an adjuvant for the hepatitis B vaccine, in increasing the immune response in HIV/AIDS patients.

## 3. Patients and Methods

The study population were all HIV/AIDS patients who had therapeutic files in the Behavioral Diseases Consultation Center affiliated to the Health Affairs Deputy of Kermanshah University of Medical Sciences. In coordination with the Health Deputy of the University, the patients who had files in the Behavioral Diseases Consultation Center in whom a HIV infection was confirmed by two positive HIV antibody (HIV Ab) enzyme-linked immunosorbent assay (ELISA) tests and one positive HIV Ab Western blot (WB) test without any history of developing hepatitis B (negative HBsAg, hepatitis B core antibody (HBcAb), and hepatitis B surface antibody (HBsAb)), and immunization against the disease were included in the study. An informed consent form was signed by all participants and the Ethical Committee of Kermanshah University of Medical Sciences approved the study. Using a table of random numbers, the participants were divided into experimental and control groups, each receiving a 40 µgr Hepavax-Gene TFvaccine(Berna Biotech,India in the deltoid muscle at 0, 1, and 3 months. Six days before each of the vaccinations and continuing until six days afterwards, patients in the experimental group received 50 mgr oral levamisole twice a day, while the controls used placebo tablets instead of levamisole, but in the same order. At the time of the second and third vaccinations and also one and three months after the third vaccination, serum samples were collected to measure the immune response. In order to reduce bias, a systematic random allocation method was used to assign study participants into experimental and control groups, as a result, all of the participants and the interviewers were blind to the allocation method. As we also had access to patients’ files, we were able to collect all of the relevant information regarding the patients’ baseline characteristics and health status history; age, sex, smoking history, and history of intravenous drug use, history of opium use, antiretroviral therapy and CD4 count. To check the efficacy of the random allocation, we compared baseline characteristics of the patients and their health profiles with a two independent samples t-test (for continuous variables) and a chi-squared test (for qualitative variables). We used a chi-squared test and two independent sample t-tests, in order to compare the level of immune response and Ab titer, respectively. The level of significance was defined as less than 0.05. Although we excluded patients who were missed during follow-up, we analyzed the data using an intention-to-treat analysis.

## 4. Results

**Figure 1:**
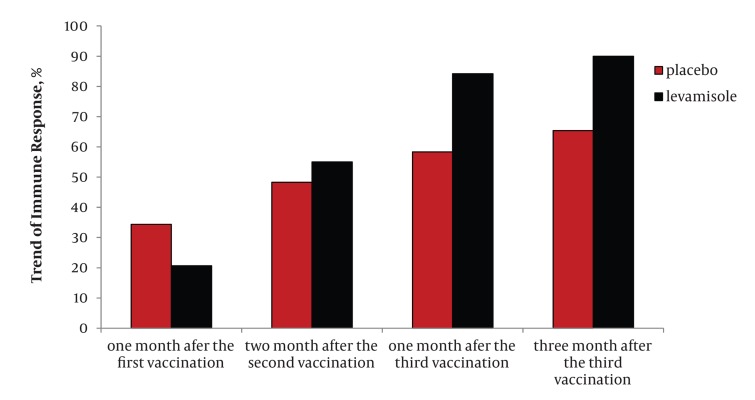
Trend of Immune Response in the Experimental and Control Groups

Among the 150 patients referred to the center during a period of 24 months (from April 2008 until April 2010), 89 patients met the inclusion criteria, and these individuals were randomly entered into the experimental (44 patients) and control (45 patients) groups, respectively. Table 1 shows the demographic data and the distribution of the variables in each of the groups. According to the table, the two groups were comparable regarding; age, sex, body mass index, history of smoking, history of using opium, history of intravenous drug use, mean count of CD4+ cells as well as antiretroviral treatment (Table 1). The second vaccination was administered to a total of 61 patients, this was because 28 individuals did not wish to continue their participation, and they did not have the second vaccination. For the third vaccination, only 49 patients received the vaccine as; four patients had died, two patients had emigrated from Kermanshah Province and six did not desire to continue their participation. Among the 49 individuals receiving all three of the vaccinations, 43 patients were referred for the first measurement of HBsAb titer, while the final (second) titration of HBsAb was performed on 46 patients. The percentage of immune response (HBsAb ≥ 10 IU/mL) and mean titer of HBsAb during the three vaccination times in the experimental and control groups are summarized in Tables 2 and 3. The level of immune response one month after the third vaccination in the experimental group (who received levamisole adjuvant) and the controls (who received placebo) showed an 84.21% and 58.33% increase, respectively. However, three months after the third vaccination, immune response had increased to 90% in the experimental group and 65.38% in the controls (P = 0.05) (Figure 1). The calculated number-needed-to treat for three months after the third vaccination is equal to 4.

**Table 1:** Demographic Data and Distribution of Study Variables in the Experimental and Control Groups

	**Levamisole Group (n = 44)**	**Placebo Group (n = 45)**	***P *value**
**Female**, %	15.9	31.11	0.09
**Age, y, Mean ± SD**	32.23 ± 7.52	6.34 ± 31.71	0.66
**BMI, kg/m^2^, Mean ± SD**	22.8 ± 2.63	22.28 ± 2.62	0.32
**Positive history of smoking, **%	84.09	80	0.61
**Positive history of using opium, **%	63.64	66.67	0.76
**Positive history of intravenous drug use, **%	72.73	71.11	0.86
**Positive history of antiretroviral treatment, **%	15.9	33.33	0.06
**CD4, **μ**L, Mean ± SD**	374.2 ± 209.6	341.46 ± 219.9	0.5

Abbreviations: BMI, body mass index

**Table 2:** Level of Immune Response During the Three Vaccinations in the Experimental and Control Groups

****	**Levamisole Group,** %	**Placebo Group, **%	***P* value**	**OR (95%CI)**
**Level of immune response one month after the first vaccination (n = 61)**	20.69	34.38	0.23	0.50 (0.16-1.60)
**Level of immune response two months after the second vaccination (n = 49)**	55	48.28	0.64	1.3 (0.42-4.1)
**Level of immune response one month after the third vaccination (n = 43)**	84.21	58.33	0.07	3.81 (0.87-16.7)
**Level of immune response three months after the third vaccination (n = 46)**	90	65.38	0.05	4.76 (0.90-25.3)

Abbreviations: OR, odd’s ratio

**Table 3:** hepatitis B Surface Antibody Titer During the Three Vaccinations in the Experimental and Control Groups

****	**Levamisole Group, Mean ± SD**	**Placebo Group, Mean ± SD**	***P *value**
**HBs-Ab titer one month after the first vaccination (n = 61)**	19.14 ± 9.07	32 ± 17.35	0.23
**HBs-Ab titer two months after the second vaccination (n= 49)**	38.85 ± 42.83	36.01 ± 39.96	0.81
**HBs-Ab titer one month after the third vaccination (n = 43)**	56.05 ± 38.16	40.12 ± 41	0.19
**HBs-Ab titer three months after the third vaccination (n = 46)**	53.75 ± 34.49	45.15 ± 38.85	0.43

Abbreviations: HBsAb, hepatitis B surface antibody

**Figure 2:**
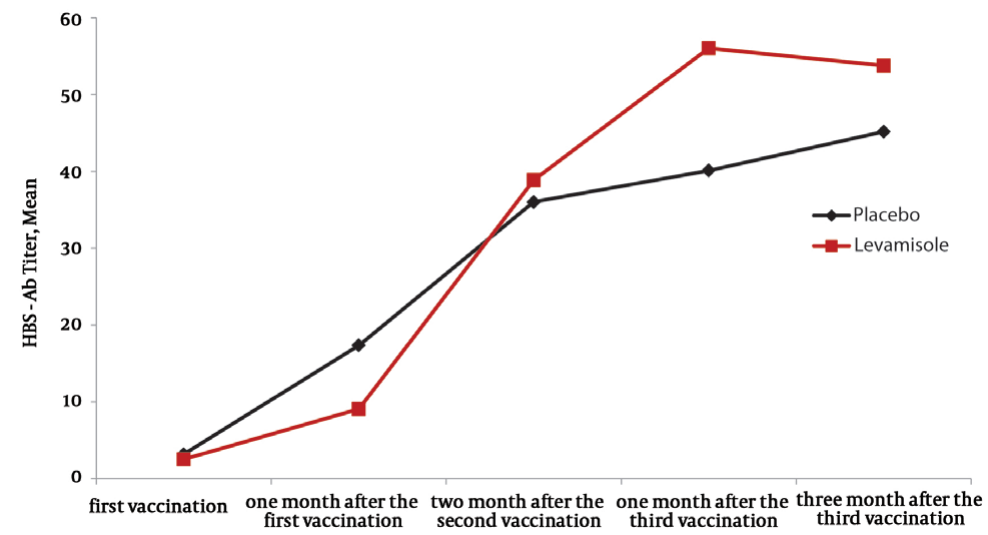
Trend of Antibody Production in the Experimental and Control Groups

As shown, the level of immune response in the experimental group was significantly higher than in the controls, and this increased with repeated vaccination. Furthermore, the mean titer of HBsAb one month after the third vaccination was 56.05 IU/mL in the experimental group and 40.12 IU/mL in the control group, and three months after the third vaccination it had increased to 53.76 IU/mL and 45.15 IU/mL in the experimental and control groups, respectively (Figure 2). According to Figure 2, the mean antibody production in the experimental group was higher than the control group. The level of immune response one month after the first vaccination was 20.69% in the experimental group, and 34.38% in the controls (OR = 0.50, 95%CI: 0.16-1.6), at the same time, the mean antibody titer was 9.07 and 17.35 in the experimental and the control groups, respectively. The level of immune response two months after the second vaccination was 55% in the experimental group and 48.28% in the controls (OR = 1.31, 95% CI: 0.42-4.11) while the antibody titer was reported to be 38.85 and 36.01 in the experimental and control groups, respectively (Figure 1 and 2). None of the variables of age or body mass index had a significant association with antibody production (P > 0.05). Moreover, the effect of the study variables including; sex, age, body mass index, history of smoking, history of opium use, history of intravenous drug use, history of receiving antiretroviral drugs, and mean count of CD4+ cells, on the trend of immune response and mean antibody titer were evaluated separately. These variables had no associations with immune response and mean antibody titer (P > 0.05), however, the mean count of CD4+ cells higher than 200 cell/mm3 significantly affected mean antibody production one month and three months after the third vaccination.

## 5. Discussion

As a result of high risk behaviors, the majority of HIV infected patients have an increased chance of developing hepatitis B (3). In addition, HBV infection in this group of patients is also associated with more complications and a higher mortality rate. Through means of inducing immunodeficiency, HIV infections result in the chronicity of HBV infection and this facilitates virus proliferation in the liver, resulting in an acceleration in and exacerbation of liver destruction (9, 10). It should be noted that the use of antiretroviral drugs and controlling opportunistic infections in HIV positive patients has resulted in longer life duration in these patients; hence, the importance of chronic infections such as hepatitis B has become more prominent. It seems that preventing HIV infected patients from developing hepatitis B, may result in a serious reduction in the complications that follow this condition and mortality rates, due to the coexistence of the two viruses. As the immune response to HBV vaccination in HIV infected patients is lower than in the normal population, the use of methods which will increase antibody production and result in a more effective immune response following vaccination, is of great importance.

As is shown in Figure 1, the administration of a levamisole adjuvant with HBV vaccination in HIV infected patients after at least two vaccinations, resulted in an increased immune response in this group of patients, in comparison with the control group. This finding is more prominent in subsequent sessions of vaccination, as the P-value of the level of immune response decreased toward the level of statistical significance. Such differences in immune response and titer of Ab, became clinically more important one and three months after the third vaccination, however, because some patients were lost to follow-up, which therefore decreases the power of the study, it did not reach a statistically significant level. There is no doubt though, that the higher immune response in the patients receiving levamisole is clinically very important, but it was not unexpected to find a statistically significant difference (P < 0.05) in immune response and antibody production in the experimental group, this may differ if the sample size was larger or the vaccination times were increased. Although using levamisole as an adjuvant for hepatitis B vaccination in HIV infected patients has been paid little attention, there are also limited studies on hepatitis vaccination and the effects of a levamisole adjuvant, inducing an immune response with other diseases. For example, in a study by Arqani et al. in dialysis patients, using a levamisole adjuvant and intradermal injection of hepatitis B vaccine was associated with a higher immune response (14). This was also the case, in a study by Sali et al. concerning the effect of levamisole on the immune response to hepatitis B vaccination in dialysis patients, it was concluded that levamisole had no effect on the level of immune response in this group of patients (19). Kayatas’ study on this issue, showed that a levamisole adjuvant increased the immune response to hepatitis B vaccination from 57% to 82% (16). In a meta-analysis by Alavian et al. on the effect of oral levamisole as an adjuvant for hepatitis B vaccine in patients with chronic renal failure, it was shown that levamisole increased seroprotection (immune protection) in this group of dialysis patients (17). In our study, patients with CD4 counts greater than 200 cell/mm3, had a substantially increased production of antibodies, one and three months after completion of vaccination. It seems that a higher CD4 count is associated with a better immune response. Thus, if the study patients in the intervention and control groups were selected from HIV infected patients with CD4 counts of more than 200 cell/mm3, it is possible that the intervention with levamisole would have had a significant impact on HBsAb production. Similarly in a study by Cruciani et al. on the immune response to higher doses and more frequent times of HBV vaccination in HIV infected patients, they excluded the patients with CD4 counts less than 200 cell/mm3 and their study showed that the patients with higher CD4 counts have better responses to higher doses and more frequent hepatitis B vaccination (20). Also in the study by Sasaki et al. the effect of granulocyte-monocyte colony-stimulating factor as the adjuvant to hepatitis B vaccine was evaluated in HIV infected patients, the patients with CD4 counts less than 350 cell/mm3 were excluded and thus a statistically significant difference between CD4 count and antibody production was not observed in the studied patients (10). Moreover, Fonseca et al. who studied a doubled dose of HBV vaccine in HIV infected patients, recognized that the patients with CD4 counts of more than 350 cell/mm3 showed a higher seroconversion level with the doubled dose, while there was no difference between vaccination dosage and immune response in the patients with CD4 counts of less than 350 cell/mm3 (12). On the other hand, Cooper et al. who studied the effect of vaccination with adjuvant CPG7909 in HIV infected patients excluded those with CD4 counts less than 200 cell/mm3 from the study and thus did not observe a significant difference in the CD4 counts between the study groups (1). In a study by Cornejo-Juarez et al. on the effects of different dosages of hepatitis B vaccine on HIV infected patients, it was concluded that because their study included patients with CD4 counts of less than 200 cell/mm3, the effect of higher doses of vaccine was not recognizable. They suggested that vaccination should be considered in HIV infected patients with a CD4 count higher than 200 cell/mm3 (9). However, it is concluded from our study that the use of levamisole in HIV infected patients is associated with a better immune response (90% in the levamisole group vs. 65.38% in the placebo group, three months after completion of vaccination), this desirable effect was more prominent in the patients with a CD4 count higher than 200 cell/mm3.

In conclusion, our study contained some limitations. The number of HIV infected patients who had not been infected by HBV and had not previously been vaccinated against HBV was small in our study. On the other hand, the number of eligible patients who left the study due to disease complications and personal and social problems was unexpectedly high, resulting in difficulties in gathering enough patients for the desirable study sample size and, hence, achieving significant statistical results. Designing multi-centric studies, as well as increasing the duration of the research, when the number of subjects is limited would increase the power of these types of studies.
